# Managing *Candida auris* fungemias: the results of a prospective and international study

**DOI:** 10.1128/aac.00358-25

**Published:** 2025-06-25

**Authors:** Hakan Erdem, Sena Şakir-Yildirim, Handan Ankarali, Ayse Batirel, Gulnur Kul, Gonca Fidan, Amani El-Kholy, Abdullah Umut Pekok, Omnia Mohamed Elnabawy Ahmed Taher, Fatma Bozkurt, Yasemin Cag, Hande Berk-Cam, Hulya Caskurlu, Esma Eryilmaz-Eren, Lutfiye Nilsun Altunal, Maha Ali Gad, Gulay Okay, Fatma Amer, Ahmet Dogan, Mehmet Emirhan Isik, Buket Erturk Sengel, Fahad Almajid, Kumar Angamuthu, Jehan ElKholy, Abu Hena Mostafa Kamal, Nilgun Karabicak, Nefise Oztoprak-Cuvalci, Souha Kanj, Pınar Yuruk-Atasoy, Berfin Cirkin-Doruk, Nagwa Mostafa El-Sayed, Nirav Pandya, Bilal Ahmad Rahimi, Elif Tukenmez-Tigen, Mustafa Uguz, Seniha Senbayrak, Canan Ağalar, Meliha Cagla-Sonmezer, Yeliz Cicek, Umran Elbahr, Ilknur Erdem, Tuba Kuruoğlu, Sibel Kuyugoz-Gulbudak, Aruna Poojary, Ertugrul Yazıcı, Tugrul Hosbul, Aysun Yalci, Hanefi Cem Gul

**Affiliations:** 1 Department of Infectious Diseases and Clinical Microbiology, Gülhane Training and Research Hospital, University of Health Sciences 448249, Ankara, Türkiye; 2 Department of Biostatistics and Medical Informatics, Istanbul Medeniyet University Faculty of Medicine 226842, Istanbul, İstanbul, Türkiye; 3 Department of Infectious Diseases and Clinical Microbiology, Dr. Lutfi Kirdar Training and Research Hospital, Health Sciences University, Istanbul, Türkiye; 4 Department of Infectious Diseases and Clinical Microbiology, Etlik City Hospital 324475 , Ankara, Türkiye; 5 Department of Clinical and Chemical Pathology, Faculty of Medicine, Cairo University 63526, Giza, Egypt; 6 Department of Infectious Diseases of Clinical Microbiology, Istanbul Aydın University Faculty of Medicine, VM Medical Park Pendik Hospital 666789 , Istanbul, Türkiye; 7 Ain Shams University Hospitals 609869, Cairo, Egypt; 8 Department of Infectious Diseases and Clinical Microbiology, Atlas University 553310, Istanbul, Türkiye; 9 Department of Infectious Diseases and Clinical Microbiology, Goztepe Training and Research Hospital, Medeniyet University 226842, Istanbul, Türkiye; 10 Department of Infectious Diseases and Clinical Microbiology, Antalya City Hospital, Antalya, Türkiye; 11 Department of Infectious Disease and Clinical Microbiology, University of Health Sciences, Kayseri, Türkiye; 12 Department of Infectious Diseases and Clinical Microbiology, Umraniye Teaching and Research Hospital, University of Health Sciences 448249, Istanbul, Türkiye; 13 Department of Infectious Diseases and Clinical Microbiology, Bezmialem University Hospital, Istanbul, Türkiye; 14 Department of Medical Microbiology and Immunology, Faculty of Medicine, Zagazig University 68799, Zagazig, Egypt; 15 Department of Infectious Diseases and Clinical Microbiology, Izzet Baysal Training and Research Hospital, Abant Izzet Baysal University, Bolu, Türkiye; 16 Department of Infectious Diseases and Clinical Microbiology, Kosuyolu Teaching and Research Hospital, Istanbul, Türkiye; 17 Department of Infectious Disease and Clinical Microbiology, Marmara University 52982, Istanbul, Türkiye; 18 Department of Medicine, King Saud University 234149, Riyadh, Saudi Arabia; 19 Department of Infectious Diseases, Almana General Hospitals 234148 , Dammam, Eastern Province, Saudi Arabia; 20 Department of Anesthesia, Pain Management, Cairo University Hospital 204584, Cairo, Egypt; 21 ICU, RMCH, Rajshahi, Bangladesh; 22 National Mycology Reference Laboratory, Public Health Directorate, Ankara, Türkiye; 23 Department of Infectious Diseases and Clinical Microbiology, Antalya Training and Research Hospital, Antalya, Türkiye; 24 Department of Internal Medicine, Division of Infectious Diseases, and Center for Infectious Diseases Research (CIDR), American University of Beirut Medical Center 66984, Beirut, Lebanon; 25 Department of Infectious Diseases and Clinical Microbiology, Bilkent City Hospital, Ankara, Türkiye; 26 Department of Infectious Diseases and Clinical Microbiology, Mersin City Hospital, Ankara, Türkiye; 27 Department of Medical Parasitology, Research Institute of Ophthalmology 155130, Giza, Egypt; 28 Bhailal Amin General Hospital 78454, Vadodara, Gujarat, India; 29 Department of Infectious Diseases, Teaching Hospital, Kandahar University Medical Faculty, Kandahar, Afghanistan; 30 Department of Infectious Diseases and Clinical Microbiology, Haydarpasa Training and Research Hospital, University of Health Sciences 448249, Istanbul, Türkiye; 31 Department of Infectious Diseases, Medicana Hospital, Istanbul, Türkiye; 32 Department of Infectious Diseases and Clinical Microbiology, Hacettepe University 539002, Ankara, Türkiye; 33 Department of Infectious Diseases and Clinical Microbiology, Medipol University, Istanbul, Türkiye; 34 Department of Infectious Diseases, Bahrain Oncology Center, Al Sayh, Bahrain; 35 Department of Infectious Diseases and Clinical Microbiology, Namik Kemal University 162334 , Tekirdağ, Türkiye; 36 Department of Infectious Diseases and Clinical Microbiology, Ondokuz Mayis University 37139, Samsun, Türkiye; 37 Department of Clinical Microbiology, Breach Candy Hospital Trust 74971, Mumbai, India; 38 Department of Medical Microbiology, Gulhane Training and Research Hospital, University of Health Sciences, Ankara, Türkiye; University of Iowa, Iowa City, Iowa, USA

**Keywords:** *Candida auris*, fungemia, bloodstream infection, antifungal, resistance

## Abstract

*Candida auris* causes hospital outbreaks and life-threatening infections, is recognized as a global health threat, and was designated a priority pathogen by the World Health Organization (WHO). Since the data on *C. auris* fungemias are quite scarce and limited to small retrospective case series, this international study aimed to prospectively assess patient characteristics, outcomes, and therapeutic approaches. The study, conducted through the Infectious Diseases-International Research Initiative (ID-IRI) platform, involved 34 referral centers. Patients with *C. auris* candidemia were prospectively enrolled between 15 April 2024 and 15 October 2024. Data on demographics, clinical and laboratory findings, treatment details, and 30-day mortality outcomes were collected. Mortality risk factors were analyzed using univariate tests and stepwise multiple binary logistic regression. The study enrolled 162 patients with a mean Charlson Comorbidity Index of 4.1 ± 2.2. Overall, 91 patients (56.2%) died. Antifungal susceptibility profiles were fluconazole (13/135, 9.6%), caspofungin (121/133, 91%), micafungin (125/126, 99.2%), anidulafungin (74/76, 97.4%), and amphotericin-B (50/134, 37.3%). Inadequate access to appropriate antifungals (odds ratio [OR] = 11.258; 90% confidence interval [CI]: 1.302–97.310; *P* = 0.065), the presence of central venous catheters (OR = 3.581; 90% CI: 1.037–12.368; *P* = 0.090), intensive care unit (ICU) stay (OR = 6.148; 90% CI: 1.977–19.123; *P* = 0.008), abdominal surgery (OR = 5.077; 90% CI: 1.651–15.610; *P* = 0.017), deep-seated candidal complications (OR = 4.546; 90% CI: 1.103–18.741; *P* = 0.079), and decreased platelet counts (OR = 1.004; 90% CI: 1.002–1.006; *P* = 0.006) were associated with increased mortality. Optimizing therapy for *C. auris* fungemia involves early strain identification, prompt echinocandin use, surveillance, proper catheter management, effective source control particularly in abdominal surgery, monitoring deep-seated candidal complications, and recognizing thrombocytopenia as a critical warning sign.

## INTRODUCTION

*Candida auris* has become a significant health issue in recent years, capable of causing deadly infections and outbreaks in hospitals (1, 2). Recognizing its critical global impact, the World Health Organization included *C. auris* in its priority list of fungal pathogens in 2022 (3). The microbe has emerged as a leading cause of hospital-acquired fungemia and is on the rise (4–6). Persistent bloodstream infections (BSIs) have been documented (7), often recurring (8), with the treatment further complicated by multidrug-resistant strains (9). Colonization or infection with *C. auris* proves exceptionally difficult to eradicate, even with targeted decolonization efforts, leaving affected patients at significant risk for developing BSIs, especially during extended hospital stays (10, 11).

Although *C. auris* is an emerging pathogen of global significance, data on *C. auris* fungemias remain scarce in the literature and restricted to small case series with retrospective data. To our knowledge, this is the first and largest prospective study on *C. auris*-related BSIs, aimed at evaluating the characteristics of patients and analyzing outcomes.

## MATERIALS AND METHODS

### Setting

The study was performed through the “Infectious Diseases – International Research Initiative” platform and involved 34 renowned referral centers, which provided data across seven countries (Türkiye, India, Saudi Arabia, Egypt, Bahrain, Bangladesh, and Afghanistan).

### Data collection

The data were prospectively collected via the Internet from 15 April 2024 to 15 October 2024, using a web-based case report form. The form captured demographic, clinical, and laboratory data; comorbid conditions; antifungal susceptibility profiles; treatment details; and 30-day mortality outcomes.

### Inclusion criteria

Patients monitored prospectively between 15 April 2024 and 15 October 2024, who had clinical findings compatible with BSIs, aged 16 years or older, and positive blood cultures for *C. auris* were included. Blood cultures were obtained at a minimum of every 48 h following the initial detection of *C. auris* in the bloodstream, continuing until subsequent negative results were confirmed (12). The treating clinicians managed the patients following international guidelines (1, 12). Accordingly, if the central venous catheter was identified as the apparent source of candidemia, it was removed at the earliest opportunity.

### Comorbid conditions

The Charlson Comorbidity Index (CCI) was used to assess the burden of comorbid conditions (13).

### Blood culture negativity

Blood culture negativity refers to the documented clearance of pathogens from the blood, as indicated by negative culture results.

### Mean arterial pressure

Arterial pressure <65 mmHg was accepted as hypotension (14).

### Fungal identification

We included matrix-assisted laser desorption ionization–time-of-flight (MALDI-TOF) mass spectrometry, Vitek2 Compact (Biomerieux, France), PCR testing, and BD PhoenixTM System (USA) as the eligible identification methods.

### Antifungal susceptibility testing

Clinical & Laboratory Standards Institute (CLSI) broth microdilution and Vitek 2 Compact were included in antifungal susceptibility testing (AFST) (15). Centers for Disease Control and Prevention (CDC) tentative breakpoints were used for AFST thresholds (16, 17). If the *C. auris* isolate was susceptible to either anidulafungin or micafungin, and the patient was treated with the other for which AFST was not conducted, the strain was considered susceptible to the administered anidulafungin or micafungin (18, 19).

### Drug resistance profiles

Multidrug resistance (MDR) was defined as resistance to two antifungal agents across the following classes: azoles (fluconazole), polyenes (amphotericin-B), and echinocandins (either anidulafungin or micafungin). Pandrug resistance (PDR) was defined as resistance to all three antifungal classes (20).

### Appropriate treatment

It was defined as a regimen containing at least one antifungal with confirmed *in vitro* activity against the *C. auris* isolate, as determined by AFST.

### Outcome

The primary outcome was defined as 30-day all-cause mortality after the initial positive blood culture for *C. auris*.

### Statistical analysis

Descriptive statistics of numerical and categorical variables were given in tables as medians, quartiles (25th, median, and 75th), numbers, and percent frequencies. The normality assumption of the numerical variables was checked with the Shapiro-Wilk test, and it was determined that they do not have a normal distribution. Simple relationships between mortality and risk factors were examined with univariate tests (Pearson *χ*^2^ and Mann-Whitney *U* test). After this step, the Stepwise Multiple Binary Logistic Regression model was used to examine the adjusted effects of risk factors on mortality. The relationship is modeled using the logit function,


(1) $$logit\left(P\right)=\ln\left(\frac{P}{1-P}\right)=b_{0}+b_{1}X_{1}+b_{2}X_{2}+b_{3}X_{3}+\ldots +b_{k}X_{k},$$

(2) $$P=\frac{1}{1+e^{logit\left(P\right)}},$$

where *P* is the probability of the dependent variable being 1 (risk).

The success of the model in mortality prediction was compared with the ROC curve and goodness of fit measures (AIC, R-square). In univariate tests, *P* < 0.05 was accepted as the statistical significance level. In our multiple model, since clinical significance and statistical significance were evaluated together, the statistical significance level was accepted as *P* < 0.10 (21). STATA/MP (ver.14.1) and JASP (ver.0.19.1.0) programs were used in the calculations.

## RESULTS

Overall, 162 patients with *C. auris* fungemia were enrolled in this study (73 [45.1%] females, median age of 66 years [range 16–96]). On the day of positive blood culture, the means of highest body temperature, mean arterial pressure (MAP), Sequential Organ Failure Assessment (SOFA) score, and CCI were 37.7°C ± 0.93°C, 89 ± 15.8 mmHg, 7.2 ± 4.9, and 4.1 ± 2.2, respectively. Ultimately, 91 patients (56.2%) died within the 30-day follow-up period. The median duration of blood culture negativity was 7 days (interquartile range [IQR] 4–14 days).

Coexisting bacterial infections: Concordant 106 infections detected in 96 (59.2%) patients were as follows: pneumonia (48 cases, 29.6%), urinary tract infections (*n* = 24, 14.8%), skin and soft tissue infections (*n* = 15, 9.3%), bacteremia of unidentified origin (*n* = 11, 6.8%), central line-associated BSIs (*n* = 3, 1.9%), intra-abdominal infections (*n* = 3, 1.9%), and bacterial meningitis (*n* = 2, 1.2%). Overall, 136 (84%) patients received antibacterial medications.

Deep-seated candidal complications in 13 patients (8%), totaling 23 complications. These included endocarditis (*n* = 9), septic embolization (*n* = 7), endophthalmitis (*n* = 3), vertebral osteomyelitis (*n* = 1), and mycotic aneurysm (*n* = 3).

Identification: Fungal identification was performed using MALDI-TOF mass spectrometry in 109 (67.3%) patients, Vitek 2 Compact (Biomerieux, France) in 42 (25.9%) patients, Vitek 2 Compact combined with PCR in 4 (2.5%) patients, and BD Phoenix System (USA) in 7 (4.3%) patients.

Antifungal susceptibility testing: AFST was conducted using broth microdilution for 49 (30.2%) isolates and Vitek 2 Compact for 85 (52.5%) isolates, while 28 (17.3%) isolates were not tested for AFST.

Antifungal susceptibility profiles: Fluconazole (13/135 [susceptible/tested], 9.6%; MIC_50_ 32 µg/mL and MIC_90_ 256 µg/mL), caspofungin (121/133, 91%; MIC_50_ 0.25 µg/mL and MIC_90_ 2 µg/mL), micafungin (125/126, 99.2%; MIC_50_ 0.12 µg/mL and MIC_90_ 2 µg/mL), anidulafungin (74/76, 97.4%; MIC_50_ 0.12 µg/mL and MIC_90_ 2 µg/mL), amphotericin-B (50/134, 37.3%; MIC_50_ 4 µg/mL and MIC_90_ 32 µg/mL), voriconazole (*n* = 62; MIC_50_ 1 µg/mL and MIC_90_ 16 µg/mL), itraconazole (*n* = 26; MIC_50_ 0.12 µg/mL and MIC_90_ 16 µg/mL), posaconazole (*n* = 22; MIC_50_ 0.12 µg/mL and MIC_90_ 4 µg/mL), and flucytosine (*n* = 52; MIC_50_ 0.5 µg/mL and MIC_90_ 64 µg/mL). The distribution of MIC values of the antifungals tested is presented in Table 1.

**TABLE 1:** Distribution of MIC values of the antifungals tested^*a*^^,^^*b*^

AntifungalDrugs	Tested	MIC values (n, %)								
		≤0.03	0.06-0.12	0.2-0.5	1	2	4	8	16	≥32
FLU	135			1, 0.7%	3, 2.2%			5, 3.7%	4, 3%	122, 90.3%
CAS	133		16, 12%	84, 63.1%	14, 10.4%	9, 6.8%	4, 3%	6, 4.5%		
MICA	126	7, 5.6%	88, 69.8%	14, 11.1%	2, 1.6%	6, 4.8%	9, 7.1%			
ANI	76	7, 9.2%	44, 57.9%	10, 13.2%	6, 7.9%	7, 9.2%	1, 1.3%	1, 1.3%		
AMB	134			13, 9.7%	27, 20.1%	23, 17.1%	25, 18.7%	15,11.2%	7, 5.2%	24, 17.9%
VORI	62	1, 1.6%	5, 8.1%	16, 25.8%	19, 30.6%	4, 6.5%	3, 4.8%	6, 9.7%	4, 6.5%	4, 6.5%
ITC	26	2, 7.7%	14, 53.8%	2, 7.7%	3, 11.5%	1, 3.8%	1, 3.8%		3, 11.5%	
POS	22	5, 22.7%	7, 31.8%	5, 22,7%	1, 4.5%		2, 9.1%	2, 9.1%		
5FC	52	1, 1.9%	21, 40.4%	6, 11.5%	6, 11.5%		1, 1.9%	1, 1.9%		16, 30.8%

^*a*^ FLU, fluconazole; CAS, caspofungin; MICA, micafungin; ANI, anidulafungin; AMB, amphotericin-B; VORI, voriconazole; ITC, itraconazole; POS, posaconazole; 5FC, flucytosine.

^*b*^ The empty cells indicate that no isolates were found with MIC values within the specified range for that column.

MDR/PDR distribution: AFST data for all three antifungal classes—fluconazole, amphotericin-B, and either anidulafungin or micafungin—were available for 125 (77.2%) isolates. MDR was detected in 73 (58.4%) patients, and PDR was detected in one case (0.8%) from a Turkish hospital in Tekirdağ.

### Therapeutic concerns

**Fig 1:**
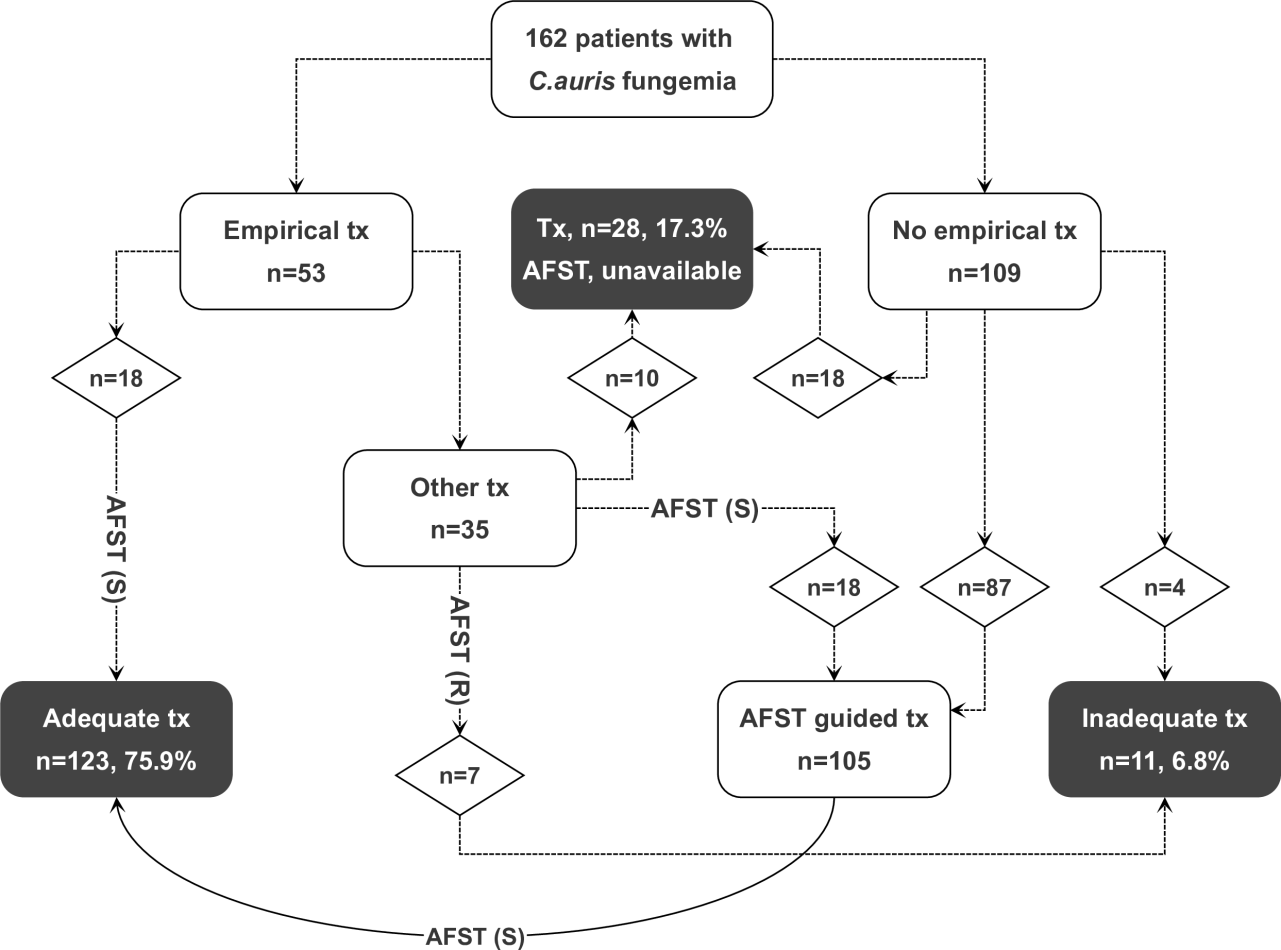
Evidence-based therapeutic approaches in the study.

- **Empirical treatment:** On the day the positive blood cultures were taken, 53 patients (32.7%) were receiving antifungal treatment: fluconazole (*n* = 27), anidulafungin (*n* = 7), caspofungin (*n* = 6), micafungin (*n* = 6), amphotericin-B (*n* = 4), and voriconazole (*n* = 3).
- **Guided antifungal treatment:** Antifungal treatment was provided based on available AFST data in 105 cases. The guided antifungal regimens included micafungin (*n* = 42), caspofungin (*n* = 49), anidulafungin (*n* = 13), and amphotericin B (*n* = 1).
- **Absence of AFST data:** AFST data were not available in 28 (17.3%) *C*. *auris* cases. In this patient group, five individuals were treated empirically with micafungin, three with voriconazole, and two with fluconazole. After the isolation of *C. auris* without available AFST data, the distribution of antifungals used was as follows: micafungin (*n* = 16), anidulafungin (*n* = 5), voriconazole (*n* = 4), caspofungin (*n* = 2), and amphotericin B (*n* = 1).
- **Appropriateness of therapy:** Empirical treatment was appropriate in 18 (11.1%) patients. Overall, 123 (75.9%) patients received adequate treatment, which was ultimately confirmed by AFST data (empirical [*n* = 18] plus guided [*n* = 105]). In 11 (6.8%) patients, adequate treatment could not be administered; 10 of these patients died before the culture data became available. One patient was started on caspofungin empirically, and despite AFST showing resistance to caspofungin, the treatment was not modified as the patient remained clinically stable and survived (Fig. 1).
- **The mainstays of antifungal treatment** were mostly echinocandins (*n* = 145, 89.5%). The distribution was as follows: micafungin (*n* = 62, 38.3%), caspofungin (*n* = 59, 36.4%), anidulafungin (*n* = 24, 14.8%), fluconazole (*n* = 7, 4.3%), voriconazole (*n* = 4, 2.5%), amphotericin-B (*n* = 3, 1.9%), and no antifungal treatment (*n* = 3, 1.9%).

### Descriptive statistics and univariate analysis

The descriptive values for the numerical characteristics of the surviving and deceased patients are presented in Table 2. In patients who died, the medians of age and SOFA score were significantly higher, median procalcitonin (PCT) values were notably elevated, and median platelet counts were significantly lower. No other factors, apart from these four characteristics listed in Table 2, were found to have a significant effect on mortality. However, only the descriptive values and univariate comparison results for C-reactive protein (CRP) and PCT were provided, and these two variables were not included in the subsequent modeling phase. The descriptive values for the categorical characteristics of surviving and deceased patients are presented in Table 3. The mortality rates were found to be significantly higher among those who could not access appropriate treatment, patients with central venous catheters, ICU patients, patients with prior hemodialysis, and those who had undergone previous abdominal surgery.

**TABLE 2:** Descriptive values for the numerical characteristics of surviving and deceased patients on the day of positive blood culture^*b*^^,*c*^

	Outcome at day 30	N	Percentiles			P^*a*^
			25th	Median	75th	
Age	Survived	71	52.00	63.00	72.0	**0.047**
	Died	91	58.00	68.00	75.0	
Charlson Comorbidity Index	Survived	71	2.00	4.00	6.0	0.136
	Died	91	3.00	4.00	6.0	
Highest body temperature (C°)	Survived	71	37.00	37.80	38.4	0.471
	Died	91	37.00	38.00	38.5	
MAP	Survived	71	80.00	89.50	100.0	0.298
	Died	91	75.00	85.00	100.0	
SOFA Score	Survived	71	2.00	5.00	8.0	**0.001**
	Died	91	5.00	8.00	10.0	
WBC	Survived	71	6910.0	11150.0	16670.0	0.617
	Died	91	6200.0	11600.0	16000.0	
Platelet	Survived	71	115000.0	179000.0	321000.0	**0.001**
	Died	91	64000.0	120000.0	200000.0	
CRP level (mg/dL)	Survived	71	12.10	60.00	168.0	0.256
	Died	91	18.00	75.00	180.0	
PCT	Survived	62	0.23	0.59	3.6	**0.013**
	Died	81	0.50	1.36	4.8	

^*a*^ Mann-Whitney *U* test. CRP, C-reactive protein; MAP: mean arterial pressure; PCT, procalcitonin; WBC, white blood cell count .

^*b*^ Bold values indicates *P*<0.05.

^*c*^ Grey layout shows the parameters of died and survived cases.

**TABLE 3:** Univariate analysis for the categorical characteristics of survivors and dead patients^*b*^^,^^*d*^

		Outcome at day 30				
		Survived (*n* = 71)		Died (*n* = 91)		
		*n*	%	*n*	%	*P^a^*
Gender	Female	36	49.3	37	50.7	0.202
	Male	35	39.3	54	60.7	
Treatment	Adequate empirical tx	8	44.4a	10	55.6a	**0.041^*c*^**
	Culture-guided tx	46	43.8a	59	56.2a	
	Inadequate tx	1	9.1b	10	90.9b	
	No AST data	16	57.1a	12	42.9a	
Antifungals	Micafungin	33	53.2a	29	46.8a	**0.037**
	Caspofungin	27	45.8a	32	54.2a	
	Anidulafungin	9	37.5ab	15	62.5ab	
	Amphotericin-B	1	33.3ab	2	66.7ab	
	Fluconazole	0	0.0b	7	100.0b	
	Voriconazole	1	25.0ab	3	75.0ab	
	No antifungal	0	0.0b	3	100.0b	
Central venous catheter	Yes	56	39.2	87	60.8	**0.001**
Total parenteral nutrition	Yes	25	36.2	44	63.8	0.093
ICU stay	Yes	51	37.5	85	62.5	**0.001**
Prior hemodialysis	Yes	6	24.0	19	76.0	**0.030**
Previous abdominal surgery	Yes	4	20.0	16	80.0	**0.022**
Weakened immune system	Yes	15	36.6	26	63.4	0.280
Diabetes mellitus	Yes	29	47.5	32	52.5	0.459
Confirmed bacterial infections	Yes	39	40.6	57	59.4	0.322
Deep-seated candidal complications	Yes	4	30.8	9	69.2	0.322
Use of antibacterials	Yes	59	43.4	77	56.6	0.794

^*a*^ Likelihood *χ*^2^ test.

^*b*^ L'a' and 'b' indicate significant differences in mortality rates between the categories of the relevant risk factor.

^*c*^ Bold values indicates *P*<0.05.

^*d*^ AST, Antifungal susceptibility data; Tx, treatment; weakened immune system: chemotherapy, organ transplantation, and neutropenia.

### Multiple binary logistic regression model

When considering the unadjusted effects of risk factors on mortality as shown in Tables 1 and 2, 10 out of a total of 22 variables were found to have significant impacts. In the subsequent stage, all 21 variables except PCT were included in the model to evaluate the adjusted effects of the risk factors. Variables without significant effects were eliminated using the stepwise variable elimination method, and those with a *P*-value < 0.10 were retained in the final model, considering their potential clinical relevance. The findings of the final logistic regression model are presented in Table 4 where the following factors were identified as significant risk factors for mortality. Inadequate access to appropriate antifungals (OR = 11.258; 90% CI: 1.302–97.310; *P* = 0.065), the presence of central venous catheters (OR = 3.581; 90% CI: 1.037–12.368; *P* = 0.090), ICUs (OR = 6.148; 90% CI: 1.977–19.123; *P* = 0.008), abdominal surgery (OR = 5.077; 90% CI: 1.651–15.610; *P* = 0.017), deep-seated candidal complications (OR = 4.546; 90% CI: 1.103–18.741; *P* = 0.079), and decreased platelet counts (OR = 1.004; 90% CI: 1.002–1.006; *P* = 0.006) were associated with increased mortality.

**TABLE 4:** Risk factors affecting mortality according to the logistic regression model

	Risk/reference	B	OR	90% CI for OR		
				Lower	Upper	*P* ^*a*^
Treatment	Culture-guided tx/adequate empirical tx	0.034	1.034	0.404	2.650	0.953
	No appropriate tx/adequate empirical tx	2.421	11.258	1.302	97.310	**0.065^*b*^**
	No AST data/adequate empirical tx	−0.511	0.600	0.198	1.823	0.450
Decreased platelet count^*a*^		−0.004	1.004	1.002	1.006	**0.006**
Central venous catheter	Yes/no	1.276	3.581	1.037	12.368	**0.090**
ICU stay	Yes/no	1.816	6.148	1.977	19.123	**0.008**
Abdominal surgery	Yes/no	1.625	5.077	1.651	15.610	**0.017**
Deep-seated candidal complications	Yes/no	1.514	4.546	1.103	18.741	**0.079**
Constant		−2.058	0.128			**0.035**

^*a*^ To estimate the model coefficients for platelet values with large magnitudes, platelet/1,000 values were used in the modeling.

^*b*^ Bold values indicates *P*<0.05.

### The performance of the model

The model demonstrates a sensitivity of 83.5%, accurately identifying deceased patients, and a specificity of 57.7%, correctly distinguishing surviving patients. The overall accuracy of the model stands at 72.2%.

## DISCUSSION

*C. auris* fungemia often presents with prolonged, difficult-to-diagnose, and hard-to-manage BSIs. The mortality rate among our fungemia patients was alarmingly high, reaching 56%. The mean peak body temperature at the time of the positive blood culture was lower than expected (37.7°C), likely reflecting the influence of the patient’s advanced age (median age 66) and co-morbid conditions (22). Moreover, the patients had substantial comorbidities, with an average CCI score of 4.1, indicating a 10-year survival probability of about 50% (23). This highlights the complex burden of *C. auris* fungemia, making it a notable challenge in critical care settings. We found that several factors in our study were significantly associated with increased mortality during *C. auris* fungemia, including limited access to appropriate antifungal therapies, the presence of central venous catheters, ICU admissions, a history of abdominal surgeries, deep-seated candidal complications, and lower platelet counts.

*C. auris* exhibits significant resistance profiles (1, 9, 24). In this study, MDR was identified in 58.4% of the isolates, posing therapeutic challenges, while PDR was observed in less than 1% of the *C. auris* strains. Close monitoring of the epidemiology is urgently needed. We found that micafungin demonstrated the highest antifungal susceptibility among the antifungals tested, which is consistent with existing literature (9, 11, 25). Overall, resistance to anidulafungin and micafungin was under 3%, while caspofungin resistance was below 10%. These findings strongly support that the echinocandin class of antifungals is the cornerstone of treatment for *C. auris* bloodstream infections, aligning with findings from other types of candidal fungemias (2, 12, 26). However, caution is indicated to monitor and address the potential development of resistance to echinocandins during treatment (27). Instead, amphotericin-B displayed resistance in two-thirds of the isolates, while fluconazole exhibited resistance in more than 90% of the isolates, significantly restricting their clinical effectiveness. Accordingly, voriconazole, itraconazole, and flucytosine demonstrated elevated MIC_90_ values, whereas posaconazole had a slightly lower MIC_90_ of 4 µg/mL. Although definite AFST thresholds have not yet been established, our data clearly indicate that non-echinocandin antifungal drugs demonstrate increased resistance profiles restricting their utility in treating *C. auris* infections. Therefore, continuous surveillance to track antifungal susceptibility patterns, along with getting hold of AFST results, is critically important.

Our data revealed that two-thirds of the patients did not receive empirical antifungal treatment, as the treating physicians did not suspect a candidal infection. Instead, 84% were prescribed antibacterial medications, underscoring that candidal BSIs are often overlooked compared to bacterial infections. Additionally, we found that the median time to fungemia clearance was 7 days, reflecting a persistent and challenging clinical course. In this study, echinocandins were the predominant therapeutic option, used in almost 90% of the cases. While inadequate access to appropriate antifungal therapies was significantly associated with increased mortality, we were unable to demonstrate a clear difference between adequate empiric and culture-guided treatments. Furthermore, the absence of AFST data did not appear to increase mortality. A few factors might explain this uniformity in outcomes. One key reason may be the prompt initiation of effective antifungal therapy—primarily echinocandins—either immediately after *C. auris* was identified or upon the laboratory reporting of yeast in the blood culture prior to fungal identification. Second, the low echinocandin resistance in this cohort may have minimized any potential benefit of tailoring treatment based on AFST results. Finally, the complexity and slower nature of fungal susceptibility testing may have eased the start of echinocandins without AFST (28). Our data underscore the importance of early identification and the role of echinocandins as a reliable first-line treatment, even in settings where AFST data are unavailable or delayed.

Hospitalizations due to *C. auris* BSIs were more commonly associated with the use of central venous catheters compared to non-fungemia infections (29). Central line-associated bloodstream infections (CLABSI) present several diagnostic challenges that complicate timely and accurate identification. A major issue is the variability in clinical symptoms and lack of specificity, leading to delays in suspicion and diagnosis, particularly when there are no local signs of infection at the catheter site. Our data indicate that the presence of central catheters is linked to higher mortality and may serve as a surrogate marker for undetected CLABSIs, underscoring the importance of early central line removal (2). Furthermore, surgery has previously been recognized as a common underlying condition in *C. auris* infections (9). To our knowledge, this is the first report that abdominal surgery has been identified as a predictor of mortality in patients with *C. auris* BSIs.

We had several limitations. First, accurately quantifying attributable mortality was challenging due to the presence of frequent comorbidities, which is the usual problem during *C. auris* infections (1, 2); therefore, we opted to use overall mortality instead. Second, phenotypic yeast identification systems, including Vitek 2, may occasionally misidentify *C. auris* strains (1, 30). However, when MALDI-TOF is unavailable, Vitek 2 is recommended as a reliable alternative for *C. auris* identification (31). Ultimately, over two-thirds of the isolates in this study were identified using MALDI-TOF, a more robust diagnostic technique (1, 30), while the rest were identified by phenotypic methods, mainly with Vitek 2. Third, the broth microdilution method is currently considered the reference standard for AFST (15). In this study, slightly more than half of the isolates were tested using the Vitek 2 Compact system. On the other hand, our study has several notable strengths. First, its prospective design integrates both laboratory and clinical data with active patient follow-up, offering a robust data set. Second, it represents the largest case series ever reported. Third, our final model shows a power of 83.5%, reflecting a high level of efficacy.

In summary, optimizing therapy for BSIs due to *C. auris*—a pathogen responsible for hospital outbreaks worldwide—involves early identification, timely echinocandin treatment, proper central venous catheter management, effective source control in abdominal surgery patients, vigilant deep-seated candidal complication monitoring, recognition of decreased platelet counts as an early warning sign, and ongoing surveillance.
